# In vitro evaluation of nano zinc oxide (nZnO) on mitigation of gaseous emissions

**DOI:** 10.1186/s40781-018-0185-5

**Published:** 2018-11-09

**Authors:** Niloy Chandra Sarker, Faithe Keomanivong, Md. Borhan, Shafiqur Rahman, Kendall Swanson

**Affiliations:** 1 Agricultural and Biosystems Engineering Department, North Dakota State University, Fargo, ND 58108 USA; 2 Animal Sciences Department, North Dakota State University, Fargo, ND 58108 USA

**Keywords:** Rumen, Feed, Greenhouse gases, Nanoparticle, Concentration

## Abstract

**Background:**

Enteric methane (CH_4_) accounts for about 70% of total CH_4_ emissions from the ruminant animals. Researchers are exploring ways to mitigate enteric CH_4_ emissions from ruminants. Recently, nano zinc oxide (nZnO) has shown potential in reducing CH_4_ and hydrogen sulfide (H_2_S) production from the liquid manure under anaerobic storage conditions. Four different levels of nZnO and two types of feed were mixed with rumen fluid to investigate the efficacy of nZnO in mitigating gaseous production.

**Methods:**

All experiments with four replicates were conducted in batches in 250 mL glass bottles paired with the ANKOM^RF^ wireless gas production monitoring system. Gas production was monitored continuously for 72 h at a constant temperature of 39 ± 1 °C in a water bath. Headspace gas samples were collected using gas-tight syringes from the Tedlar bags connected to the glass bottles and analyzed for greenhouse gases (CH_4_ and carbon dioxide-CO_2_) and H_2_S concentrations. CH_4_ and CO_2_ gas concentrations were analyzed using an SRI-8610 Gas Chromatograph and H_2_S concentrations were measured using a Jerome 631X meter. At the same time, substrate (i.e. mixed rumen fluid+ NP treatment+ feed composite) samples were collected from the glass bottles at the beginning and at the end of an experiment for bacterial counts, and volatile fatty acids (VFAs) analysis.

**Results:**

Compared to the control treatment the H_2_S and GHGs concentration reduction after 72 h of the tested nZnO levels varied between 4.89 to 53.65%. Additionally, 0.47 to 22.21% microbial population reduction was observed from the applied nZnO treatments. Application of nZnO at a rate of 1000 μg g^− 1^ have exhibited the highest amount of concentration reductions for all three gases and microbial population.

**Conclusion:**

Results suggest that both 500 and 1000 μg g^− 1^ nZnO application levels have the potential to reduce GHG and H_2_S concentrations.

## Background

The agricultural sector is recognized as one of the greatest sources of methane (CH_4_) and other gaseous emissions, and it is contributing approximately 250 million metric ton CO_2_ Eq. CH_4_ emission per year [1, 2]. Most of the CH_4_ emissions from the agricultural sector are from the livestock industry and manure management. Almost 70% of the agricultural sectors CH_4_ emission is from enteric fermentation [3]. Enteric fermentation includes fermentation in the rumen and hindgut paired with digestive hydrogen (H_2_) metabolism by microbial catalyst [1]. During enteric fermentation, CH_4_ and carbon dioxide (CO_2_) are the two main greenhouse gases (GHGs) emitted and contribute to global warming [1]. Hydrogen sulfide (H_2_S) is another pollutant gas generated during enteric fermentation, although its amount is not significant compared with CH_4_ and CO_2_. Hydrogen Sulfide might be a potential health hazard to livestock and workers depending on the concentration level [4]. Hence, the reduction of these gas emissions without altering animal productivity is a challenge for a healthy environment and sustainable livestock industries.

Fermentation of carbohydrates in the reticulorumen occurs for available hydrogen supply towards volatile fatty acid (VFA) production and eventually leads to CH_4_ production [5–9]. Additionally, fermentation and neutralization of hydrogen ions (H^+^), and bicarbonate ions (HCO^3−^) entering the rumen across the ruminal wall during VFA absorption contributes to CO_2_ production in the rumen [10, 11]. Similarly, sulfur-containing amino acids and sulfates are the main sources of H_2_S within the rumen; H_2_S generation depends on the microbial degradation of amino acids and sulfates [11–13].

Since all of these gaseous emissions pose potential environmental and safety concerns, scientists are striving to mitigate the production of these gases. Management of feeding strategy, application of biotechnology, and the introduction of additives are a few of the most common approaches that researchers are working on for abating enteric gaseous emissions [14]. Similarly, changes in the forage species, good forage processing, reduction of forage maturity, based on your excellent credentials, and increased feeding frequency are a few noteworthy gas mitigation strategies [14–21]. However, all of these approaches exhibit a very small amount of gaseous emission reduction, and in most of the cases, the mitigation strategy focused on the reduction of CH_4_ only. So, it is important to develop a new approach that can reduce multiple gaseous emissions without compromising animal health and productivity.

In recent years, nanotechnology has received attention for improving livestock production [22]. In U.S., only 26 of 160 agri-food nanotechnology research and development projects were relevant to livestock facilities [22]. Animal health, veterinary medicine, and other animal production facilities are a few of the livestock-related sectors on which nanoparticles (NPs) have their promising footprints [23–25]. For example, silver and zinc NPs have been added to animal feed to control microbial proliferation and promote animal growth, respectively. Similarly, zinc oxide (nZnO) NP is used to enhance growth and feed efficiency in piglets and poultry [26]. However, application of nanotechnology in mitigating gaseous emissions from livestock facilities is still limited. Swain et al. [26] reported nZnO changes the rumen fermentation kinetics in ruminants and can alter the volatile fatty acids, therefore it may affect enteric CH_4_ production. Similarly, application levels of NPs may also alter the microbial population, thus other gaseous emissions. Among the few studies performed with GHGs mitigation, nZnO were reported to have an inhibitory action towards CH_4_, CO_2_ and H_2_S from anaerobic storage of manure [27, 28]. Therefore, the objective of this study was to evaluate the efficacy of four different application rates (100, 200, 500, and 1000 μg g^− 1^ of feed) of nZnO in mitigating CO_2_, CH_4_, and H_2_S emissions from rumen fluid under anaerobic storage conditions. Other than the application rate of 1000 μg g^− 1^, nZnO application rates were within the general dietary guideline of the maximum tolerable level of Zn mineral concentration provided by the National Academies of Sciences [29]. The specific objective was to characterize the changes in the rumen fluid properties and find the gaseous reduction mechanisms such as by bacterial population reduction.

## Methods

### Ruminal fluid collection, processing and experimental setup

Ruminal fluid was collected from two ruminally-fistulated mature steers predominately of Angus breeding on a limit-fed grass hay-based diet fed to maintain body weight. Two hours after morning feeding, approximately one liter of ruminal fluid was collected from each steer. To ensure uniform representation of the liquid and fiber phase, random grab samples were collected both from ventral and dorsal ruminal sacs. Prior to mixing with McDougall’s buffer [30], ruminal fluid from each steer was combined and strained through four layers of cheesecloth to remove the large particulate matter. Five treatments consisting of a control (no nZnO) and four levels of nZnO (100, 200, 500, and 1000 μg g^− 1^ of feed), with two different feeds (alfalfa and maize silage; Table 1**)** were used. Nutrient compositions of the two base diets are shown in Table 1. Levels of nZnO were selected based on the maximum allowable zinc (Zn) concentration (30 to 500 μg g^− 1^) in feed recommended by the [29]. The 1000 μg g^− 1^ of nZnO level was added to investigate the effect of high nZnO application level on ruminal gaseous emission. The nZnO application levels were weighed on a Sartorius CP2P microbalance (Sartorius Corporation, NY, USA) with an accuracy of 1 μg using small aluminum pans (DSC Consumables*,* Inc., AU, USA). The nZnO (US Research Nanomaterials, Inc., Texas, USA; Particle Size = 35–45 nm and 99.5% purity) was mixed with two feeds (e.g., alfalfa and maize silage) separately. In each ANKOM^*RF*^ gas bottle, 1.5 g of ground alfalfa or maize silage (3 to 5 mm size) feed was added. Thereafter, 37.5 mL of the combined rumen fluid and 150 mL of McDougall’s buffer were added to each bottle and a sub-sample of the mixed ruminal fluid was stored in the freezer for characterization. Treatment bottles was purged with CO_2_ to create an anaerobic environment and sealed with the ANKOM^*RF*^ pressure monitor cap. Thus, in total, twenty (5 treatments × 4 replications) bottles were used for each feed type.

**Table 1:** Composition of the feeds (dry matter basis)

Feeds	%									
	Ash	CP	NDF	ADF	Ca	P	Mg	K	Zn	Cu
Alfalfa	13.16	18.33	60.28	42.59	3.99	0.29	0.39	3.26	0.01	0.06
Maize silage	7.06	6.02	53.65	31.42	0.88	0.26	0.20	1.37	0.01	0.08

*CP* Crude Protein, *NDF* Neutral Detergent Fiber, *ADF* Acid Detergent Fiber, *Ca* Calcium, *P* Phosphorus, *Mg* Magnesium, *K* Potassium, *Zn* Zinc, *Cu* Copper

### Ruminal pH and redox determination

The pH, and redox of the mixed ruminal fluid were determined before and after the ruminal fluid was treated with nZnO using a HANNA HI 4522 dual channel benchtop meter (VWR, TX, USA). Both probes were calibrated following manufacturer standard protocols. The reading of each probe was also confirmed with respective standard solutions before each measurement to ensure accurate reading of the probes.

### Gas production measurement and monitoring system

All experiments were conducted using 250 mL ANKOM^*RF*^ gas glass bottles and under the same conditions. After proper flushing and sealing of bottles, they were placed in a water bath (SWBR17 shaking water bath, Atkinson NH, USA) that oscillated and heated at 125 rpm and 39 ± 1 °C, respectively. Once they were placed in the water bath, a wireless gas production measurement system **(**ANKOM Technology Corp., Macedon, NY, USA) was used for monitoring and measuring gas production data. Data obtained from this system were converted from pressure (KPa) units to volume units (mL) using the ideal gas law as follows:

$$ n=p\left(\frac{V}{RT}\right)\kern12em Eqn\ 1 $$

$$ Gas\  produced\ (mL)=n\times 22.4\times 1000\kern1em Eqn\ 2 $$

Where: n = gas produced in moles (mol), P = pressure in kilopascal (kPa), V = head-space volume in the Glass Bottle in Liters (L), T = temperature in Kelvin (K), and R = gas constant (8.314472 L.kPa.K^− 1^.mol^− 1^).

Throughout the experimental period, Each bottle was connected to a Tedlar bag and once gas pressure inside a bottle reached a set-limit in the RF pressure sensor module and recorded by the ANKOM^*RF*^ system, the headspace gas was released in the connected Tedlar bag. A typical in vitro study lasts for 24 h, however, in the present study it was continued for 72 h to examine the effects of nZnO on long term in vitro fermentation. After 72 h of the experimental period, gas samples from the Tedlar bags were drawn using a gas-tight syringe (5 mL, Luer-LokTM Tip Syringe, Franklin Lakes, NJ, USA) and analyzed for GHGs (CH_4_ and CO_2_), and H_2_S concentration. A Jerome Meter (Jerome 631X, Arizona Instrument LLC, Arizona, USA) was used to measure H_2_S concentration and a gas chromatograph (GC, 8610C, SRI instrument, California, USA) equipped with flame ionization detector (FID) and electron capture detector (ECD) detectors were used to measure CH_4_ and CO_2_ concentration. Based on the previous trials, collected gas was diluted 100 fold with pure nitrogen to keep the concentration in the measurable range of the analytical instruments and two measurements for individual bottle were taken for each of CH_4_, CO_2_, and H_2_S concentration. Nitrogen at 20 psi with a flow rate of 250 mL min^− 1^ was supplied to the GC as a carrier gas. Additionally, a built-in air compressor and external hydrogen generator were used to supply hydrogen and air to the GC. Temperatures of 300 and 350 °C were maintained respectively on the FID and ECD detectors before insertion of any sample gas into the GC sample loop [31]. Calibration gases were used to check the proper functioning of the instruments and blank samples were used to check any contamination within the instruments from previous measurements [32].

### Analysis of microbial populations

Rumen fluid samples ( ~ 5 mL) were collected at the beginning (just before the experiment) and at the end of the experiment (after 72 h of the experiment) and they were analyzed for the coliforms i.e. potential pathogens (particularly *Escherichia coli*) that is recommended by the American Public Health Association (APHA) and the Environmental Protection Agency (EPA). Microbial populations (coliforms) density were analyzed by counting total coliform bacteria in terms of colony forming units (CFUs) following the plate count method [33]. All reagents, labware, and Petri dishes used for microbial analysis were handled carefully and the whole experimental preparation was conducted in a sterile environment. One milliliter of the rumen fluid samples were collected from each treatment replications, and were diluted up to five-fold (10, 10^2^, 10^3^, 10^4^ and 10^5^) to find the optimum dilution for better visibility of the CFUs. Later on, all treatments were replicated three times with the optimum dilution. The 2 mL M-Endo broth ampule (P/N: 23735–50, HACH LANCH GmbH, Willstatterstrasse 11, Dusseldorf, Germany) was used as growth media to culture the bacteria in an incubator. The growth media was poured evenly over a gridded sterile membrane filter attached with absorbent pad (47 mm diameter, 0.45 μm pore size, WCN type, Whatman Limited, Maidstone, England, UK) that was placed in a sterile petri-dish (Anaerobic, Sterile Petri dishes, 60 mm diameter and 15 mm height, VWR, Radnor, PA, USA). Subsequently, 100 μL of the diluted rumen fluid samples were added to the absorbent pad and smeared evenly over the pad using a small sterile glass rod. The petri dishes with the growth media and bacterial culture were then incubated for 24 h at 35 ± 0.5 °C in an incubator (Lab Companion IB-01E Incubator, San Diego, CA, USA). After 24 h of incubation, CFUs were counted using a manual dark field colony counter with 1.5X magnification (Reichert, Inc. Depew, NY, USA).

### Volatile fatty acids (VFAs) analysis

At the end of the experimental period, Whirl-Pak bags (Nasco, Fort Atkinson, WI and Modesto, CA, USA; 532-mL) were used to collect and store the rumen fluid subsamples at − 20 °C until further analysis. Thereafter, samples were equally composited using a vortex (Cat: 10153–842, VWR® digital vortex mixer, Radnor, PA, USA) and centrifuged (clinical 100 laboratory centrifuge, VWR, Rndor, PA, USA) at 2000×g for 20 min. They were filtered through a pore size 0.45 μm to separate out the supernatant and analyzed for VFAs using an Agilent 6890 N gas chromatograph (Agilent Technologies, Inc., Wilmington, DE, USA) equipped with an FID and fused silica column (Supleko brand, NUKUL 15 m × 0.53 mm × 0.5 μm, Sigma-Aldrich C., MO, USA), and 7683 series auto-injector following a widely used method [34].

### Statistical analysis

The data were analyzed in a 2 × 2 factorial experiment using PROC GLM (SAS Inst. Inc., Cary, NC), which calculated the statistics for general linear models. Both of the feed types and five levels of nZnO were used as fixed effects models. Means were declared statistically significant at *P* ≤ *0.05* using Duncan multiple range test.

## Results

### Effect of nZnO application levels on ruminal pH and redox

The pH of the rumen fluid incubated 72 h with alfalfa ranged between 7.20 to 7.25, whereas the pH of the maize silage based rumen fluid ranged between 6.92 to 6.96 (Table 2). Alfalfa based rumen fluid showed significantly higher pH than of maize silage *(P < 0.0001)*. No interaction was found between feed types and nZnO levels (*P = 0.401*). Additionally, none of the zinc levels (100 to 1000 μg g^− 1^) were found to indicate a significant difference in pH values *(P = 0.644).* Redox potential among the treated rumen fluid and two different feed combinations ranged between − 296 to − 307 mV (Table 2), which is the preferred range for producing CH_4_ and CO_2_ anaerobically [35]. The rumen fluid redox potential between two feed types were not significantly different *(P = 0.748)*. Additionally, similar to that of pH, no interaction among the feed types and nZnO levels was found for the rumen fluid redox potential *(P = 0.217),* and no significant difference was found among the nZnO levels *(P = 0.947).*

**Table 2:** Effect of nZnO levels on ruminal pH and redox (after 72 h of incubation)

Effects	Alfalfa	Maize silage	Control	100 μg g^−1^	200 μg g^− 1^	500 μg g^− 1^	1000 μg g^− 1^	SEM^a^	*P* value		
									Feed	nZnO	nZnO^a^ Feed
pH	7.22x	6.94y	7.09a	7.07a	7.08a	7.07a	7.08a	0.001	<.0001	0.644	0.401
Redox	-300x	-301x	-301a	-301a	-300a	-302a	-299a	9.004	0.748	0.947	0.217

^a^Data’s are presented as least square means per treatment ± SEM

Means followed by the same letters (x/y/a/b/c/d) in each row are not significantly different at *P ≤ 0.05*

### Effect of nZnO application levels on ruminal VFA production

Among the four nZnO levels and the control treatment, the amount of total VFA (TVFA) ranged between 136.52 to 194.16 mM for the alfalfa-based rumen fluid, while it ranged between 161.36 to 192.8 mM for the maize silage based rumen fluid**.** Compared with the other treatments (nZnO levels), after 72 h of the experimental period, the control treatments exhibited the highest TVFA (Table 3). For the acetic acid, no significant difference was found among the feed types *(P = 0.832),* and no significant interaction between feed types and nZnO levels *(P = 0.172)* was found. Moreover, no significant interaction between feed type and nZnO concentrations (*P* = 0.688) were found for the propionic acid. However, rumen fluid with alfalfa had significantly lower propionic acid concentration than rumen fluid with maize silage *(P < 0.001)*. Propionic acid was also found to be affected by nZnO levels (Table 3), although, no definite trend was found. Propionic acid to acetic acid (P/A) ratio was higher for maize silage based fermentation than the alfalfa-based fermentation. Alfalfa-based rumen fermentation’s P/A ratio varied from 0.29 to 0.32, whereas this ratio varied from 0.38 to 0.55 for the maize silage-based fermentation.

**Table 3:** Effect of nZnO levels on the rumen fluid VFA (*n* = 4 observations/treatment)

Effects	Alfalfa	Maize silage	Control	100 μg g^−1^	200 μg g^− 1^	500 μg g^− 1^	1000 μg g^− 1^	SEM^a^	*P* value		
									Feed	nZnO	nZnO^a^ Feed
Acetic Acid (mM)	108x	107x	122a	97.1b	98b	108ab	114a	14.15	0.832	0.005	0.172
Propionic Acid (mM)	33.05x	49.53y	50.88a	34.01c	36.94bc	41.11bc	43.50ab	7.68	<.0001	0.002	0.688
P/A ratio	0.306x	0.444y	0.423a	0.389a	0.369a	0.379a	0.380a	0.058	0.189	0.004	0.007
Total VFA (mM)	161x	173x	193a	149c	152bc	166bc	174ab	21.51	0.086	0.002	0.743

^a^Data are presented as least square means per treatment ± SEM

Means followed by the letters (x/y/a/b/c/d) in each VFA type are not significantly different at *P ≤ 0.05*

*VFA* Volatile Fatty Acid, *P/A* Propionic acid to Acetic acid ratio

### Effect of nZnO application levels on ruminal gaseous emission and CH_4_, CO_2_, and H_2_S concentrations

Table 4 represents the amount of total gas produced, and gas concentrations in the ANKOM^*RF*^ bottles over 72 h of incubation with four different nZnO application levels and two feed types. Produced total gas from the maize silage fermentation was two times higher than that of alfalfa fermentation *(P < .0001).* However, no significant difference in terms of total gas production among different applied nZnO levels was found *(P = 0.875)*. Moreover, there was no significant interaction between feed types and zinc levels were evidential *(P = 0.542).*

**Table 4:** Effect of nZnO levels on cumulative gas volume and gas concentrations (*n* = 4 observations/treatment)

Effects	Alfalfa	Maize silage	Control	100 μg g^− 1^	200 μg g^− 1^	500 μg g^− 1^	1000 μg g^− 1^	SEM^a^	*P* value		
									Feed	nZnO	NZnO ^a^Feed
Total Gas (mL)	41.09x	72.77y	58.17a	55.88a	55.88a	57.99a	58.17a	5.71	<.0001	0.875	0.542
CH_4_ (%)	10.69x	9.47y	12.68a	11.52ab	10.18bc	9.26c	6.74d	1.44	0.0114	<.0001	0.479
CO_2_ (%)	48.78x	45.55y	56.21a	53.46ab	50.06b	43.93c	32.16d	4.51	0.0314	<.0001	0.948
H_2_S (ppm)	3420x	1314y	3150a	2856a	2431b	1936c	1460d	298	<.0001	<.0001	<.0001

^a^Data are presented as least square means per treatment ± SEM

Means followed by the letters (x/y/a/b/c/d) in each row are not significantly different at *P ≤ 0.05*

Measured total gas volume from the maize silage based rumen fluid was significantly higher than that of alfalfa based rumen fluid *(P < .0001)*, although maize silage based rumen fluid produced lower CH_4_, CO_2_, and H_2_S gas concentrations than that of alfalfa based rumen fluid. However, all of the nZnO levels irrespective of the feed types showed a similar reduction trend for both CH_4_ and CO_2_ concentrations. Regardless of the feed and nZnO levels, CO_2_ concentrations were around five times higher than that of CH_4_ concentrations. In contrast, although the evidential significant interaction between feed types and zinc levels *(P < .0001)* was found for H_2_S concentration (Table 4), but the reduction trends were similar to that of CH_4_ and CO_2_. H_2_S concentration from the maize silage was ~ 60% less than that of alfalfa. Regardless of the feed used, compared to control treatment higher nZnO application levels reduced higher amount of CH_4_, CO_2_, and H_2_S concentrations. Compare to the control, pooled average of the gas concentrations showed that applied levels of nZnO reduced CH_4_, CO_2_, and H_2_S concentration by 9.14 to 46.85%, 4.89 to 42.79%, and 9.33 to 53.65%, respectively. Among the treatments, the 1000 μg g^− 1^ of nZnO application level produced the highest reduction in CH_4,_ CO_2_, and H_2_S concentration *(P < .0001*). Additionally, both 500 and 1000 μg g^− 1^ nZnO levels reduced significant *(P < .0001)* amount CO_2_ and H_2_S concentrations compared to other treatments (Table 4). However, no significant interaction between feed level and zinc was found for CH_4_ *(P = 0.479)* and CO_2_ *(P = 0.948).*

### Effect of nZnO application levels on ruminal microbial population

Plate counts were done in terms of CFUs from pre- and post-treated rumen fluid samples to determine the effects of applied nZnO on coliforms (Table 5). The average initial CFUs were 88.4 counts with alfalfa feed based rumen fluid, and it was 85.2 counts with the maize silage feed based rumen fluid. Initial CFUs were similar regardless of feed types *(P = 0.231)* or nZnO inclusion *(P = 998)*. In contrast, final CFU numbers exhibited a different trend than the initial number of CFUs (Table 5). At the end of the 72 h experimental period, CFU numbers increased by ~ 98% for all of the treatments including control, and they ended up with an average of 4630, and 5155 counts for alfalfa and maize silage feeds, respectively. Irrespective of the nZnO application levels, final CFU counts were higher with maize silage compared with the alfalfa. Although, lower application levels of nZnO exhibited very small CFU reduction efficiency compared with the higher levels. The greatest reduction in microbial population was observed at the highest nZnO level.

**Table 5:** Effect of nZnO levels on ruminal microbial populations (n = 4 observations/treatment)

Effects	Alfalfa	Maize silage	Control	100 μg g^− 1^	200 μg g^− 1^	500 μg g^− 1^	1000 μg g^− 1^	SEM^a^	*P* value		
									Feed	nZnO	nZnO ^a^Feed
Initial_CFU	88.40x	85.20x	87.63a	86.50a	86.38a	86.88a	86.63a	8.27	0.231	0.998	0.923
Final_CFU	4630x	5155y	5350a	5325a	4900a	4725ab	4162b	590	0.009	0.002	0.887

^a^Data are presented as least square means per treatment ± SEM

Means followed by the letters (x/y/a/b/c/d) in each row are not significantly different at *P ≤ 0.05*

*CFU* Colony Forming Unit

## Discussions

Lower pH of the rumen fluid incubated with maize silage based treatments might affect/inhibit acidogenic bacteria those are responsible for anaerobic digestion. In contrast, higher pH in alfalfa feed based treatments might increase the rate of fermentation, and contribute to the growth of spoilage microbes [36–38]. Moreover, the higher pH in the post-treated alfalfa-based rumen fluid would likely produce a higher amount of soluble protein, carbohydrate, and volatile fatty acids [39]. Hence, higher concentrations of all three gases (CH_4_, CO_2_, and H_2_S) were likely from the alfalfa based treatments compared with its counterpart. The resulted consistent redox potential among the treatments was preferred for anaerobic fermentation [40–43]. Additionally, redox potential among the treated rumen fluid and two different feed combinations were in the preferred range for producing CH_4_, CO_2_, and H_2_S anaerobically [44].

Volatile fatty acids are considered as one of the most important parameters for ensuring anaerobic fermentation. Higher TVFA amount in maize silage based rumen fluid compared with the alfalfa forage types might be an indication of the higher amount of digestible carbohydrate in the maize silage [45]. Subsequently, a higher amount of cumulative gas production from the maige silage-based fermentation was likely. The resulted pooled average of P/A ratio in the present study was 0.304 and 0.459 for the alfalfa and maize silage, respectively. The P/A ratio from the maize silage was 26% higher than the previously reported value, while the P/A ratio for the alfalfa was identical to the reported value (Ghimire, 2015). Higher P/A ratio might be an indication of imbalanced anaerobic fermentation with the maige silage-based rumen fluid fermentation [42]. Application of nZnO was hypothesized to affect either hydrolysis, acetogenesis, fermentation, methanogenesis or a combination of these processes in the fermentation process. In some cases, the bactericidal action of the applied higher nZnO level might kill the higher amount of methanogens, and hence a higher amount of unconverted TVFA was likely. Furthermore, increased energy utilization followed by ruminal microbial protein synthesis by the microbes in the early stages of fermentation might have increased the TVFA with the applied higher nZnO level as indicated by others [46].

Higher gas production from the maize silage fermentation might be due to probable higher carbohydrate content and subsequent higher fermentability of maize silage compared to alfalfa. None of the applied nZnO application levels were able to reduce total gas volume significantly, even 1000 μg g-1 of nZnO was not enough to reduce a significant amount of cumulative gas production. Therefore, nZnO at this application rate does not appear to decrease the digestibility of feed by the animal, and therefore, should not decrease productivity or growth. However, further studies are needed to understand the process and to verify if the productivity is sustained when nZnO is included in the diet.

It is noteworthy that CH_4_ concentrations with alfalfa were higher than those of maize silage (Table 4), although higher cumulative gas production was observed in maize silage-based fermentation (Table 4). This was likely due to appropriate P/A ratio and subsequent balanced fermentation with alfalfa-based rumen fluid that might prompt higher CO_2_ and H_2_S concentration as well [42]**.** Generally, a group of archaea belonging to the phylum *Euryarcheota*, and collectively known as methanogens are responsible for CH_4_ production within the animal rumen and hindgut [47]. Reduction of the CH_4_ concentration from rumen fluid at the highest application level of nZnO was likely due to the impact of excessive nZnO application rate (which was almost two-fold of the allowable limit as recommended by NAS as feed) specifically on methanogens [26]. As mentioned previously, the highest application rate (1000 μg g^− 1^) of nZnO did not affect total gas production, but likely reduced the enteric CH_4_ concentration due to inhibitory action on the CH_4_-producing methanogenic microbial community. Additionally, adsorption of the produced methane on the NPs surface might also contribute to the reduction in CH4 when nZnO was added to the rumen fluid. This situation warrants further study for investigating the effect of higher levels of zinc as a feed additive on animal growth and productivity.

CO_2_ concentration was five times higher than the CH_4_, which might be an indication of biocidal action of nZnO on methanogen archaea. During anaerobic digestion process, methanogenic archaea utilize CO_2_, and H_2_ to produce CH_4_. Nano zinc oxide might leave only a small amount of methanogenic archaea active, and thus higher amount of unconverted CO_2_ was likely. Furthermore, CO_2_ emission from rumen is directly related to the degradation of the organic constituents present in the feed, hence the decreasing trend in the CO_2_ concentration was likely to indicate lower degradation rate of the organic matter in the rumen. Application of NPs might have an adverse impact on the microbial community and as a consequence lower degradation of organic compounds might occur. However, additional microbial studies are needed to understand the in-depth process.

The Higher amount of H_2_S concentration from the alfalfa based feed compared with the maize silage was likely to be an indication of higher activity of the microorganisms. Since, in absence of oxygen (O_2_) sulfate-reducing bacteria utilize sulfate to oxidize organic compounds present in the feed and ends up with the H_2_S production as a byproduct, hence the reduction trend of H_2_S concentration might be due to the reduced activity of the sulfate-reducing bacteria [48]. However, the concentration reduction mechanism needs to be explored to investigate the adverse effect of the nZnO on the microbial community.

Initial CFUs were measured right after the application of the nZnO in the system, therefore, there was little or no effects of nZnO levels on CFUs. In this circumstance, irrespective of the nZnO application levels, the number of microbial populations was most likely to represent the similar number of the populations present in the rumen fluid. In contrast, addition of fresh feed was most likely to contribute towards the increasing amount of final CFUs. Compared with the control (final), lower CFU numbers in the nZnO treated samples were most likely due to the biocidal effect of nZnO. An insignificant amount of CFUs reduction from the treatments with lower application levels of nZnO might be an indication of the lower amount of available biocides. In contrast, a higher reduction in CFUs was observed with higher application levels of nZnO and the reduction was significant only with 1000 μg g^− 1^ inclusion level. Furthermore, the presence of higher CFUs in the maize silage based treatments were likely to validate the higher gas production from those treatments, and vice versa. Additional study at different levels and feed types are needed to understand in depth CFU reduction chemistry of nZnO.

## Conclusions

Within the same feed type, application of nZnO has no impact on the rumen fluid pH, and redox potential. Compared with the control treatment, higher nZnO application levels (500 and 1000 μg g^− 1^) reduced CH_4_, CO_2_ and H_2_S concentrations significantly (ranged from 21.85 to 53.65%). Similarly, the 1000 μg g^− 1^ inclusion level reduced the microbial population in both feeds significantly (22.21%) as compared to control treatment. Based on this study, the inclusion of 500 or 1000 μg g^− 1^ nZnO may reduce enteric fermentation resulting in lower enteric GHG emission from grass fed beef. However, additional microbial studies are necessary to determine the mode of action. Additionally, further work is needed to assess the effect of nZnO inclusion on animal performance when cattle are fed ingredients commonly used in beef feedlot diets.
